# The Effect of Polyaniline (PANI) Coating via Dielectric-Barrier Discharge (DBD) Plasma on Conductivity and Air Drag of Polyethylene Terephthalate (PET) Yarn

**DOI:** 10.3390/polym10040351

**Published:** 2018-03-22

**Authors:** Shuai Liu, Deqi Liu, Zhijuan Pan

**Affiliations:** 1 College of Textile and Clothing Engineering, Soochow University, Suzhou 215021, China; liushuai@suda.edu.cn; 2 College of Chemistry, Chemical Engineering and Materials Science & Collaborative Innovation Center of Suzhou Nano Science and Technology, Soochow University, Suzhou 215123, China; 3 Suzhou Key Laboratory of Green Chemical Engineering, Soochow University, Suzhou 215123, China

**Keywords:** polyaniline (PANI), DBD plasma, conductive PET yarn, air drag, volume resistivity

## Abstract

In this paper, a simple method to prepare PANI-coated conductive PET yarn is reported, which involves pre-applying aniline and HCl vapors on PET surface and subsequent dielectric-barrier discharge (DBD) plasma treatment of the coated yarn under atmospheric pressure. The volume resistivity of the optimal sample was about 1.8 × 10^5^ times lower than that of the control. Moreover, with the increase of coating amount of PANI, the air drag of PET yarns improved gradually. The surface chemistry of the treated yarn was analyzed by Fourier transform-infrared (FT-IR) spectroscopy and X-ray photoelectron spectroscopy (XPS), while the morphology was observed by scanning electron microscopy (SEM) and atomic force microscopy (AFM). This study offers a new method to prepare conductive fabric via air-jet loom and is expected to increase the weaving efficiency of air-jet loom.

## 1. Introduction

Polyethylene terephthalate (PET) fiber is one of the synthetic fibers with the largest yield in the world, and possesses excellent mechanical and bulk properties [1,2]. However, owing to its hydrophobic character, PET fiber easily accumulates static charge. At present, different methods have been employed to produce conducting fabrics, such as fiber modification, fabric anti-static treatment, and including evenly spaced metal filaments in the fabric [2]. The synthesis of intrinsically conducting polymers (ICPs) on PET fiber is an efficient way to improve the conductivity of PET fiber. Among the common ICPs, polyaniline (PANI) has been extensively studied because of its low cost, ease of synthesis, good electrical property, chemical and environmental stability, and the ability to be electrically switched between its conductive and resistive states [3,4,5]. However, the processability as well as the mechanical and electrical properties caused by the high conjugated structure in the molecules and strong intermolecular interaction of PANI need further improvement. Therefore, its applications are limited, and there is a lack of flexibility [6,7,8,9]. Since the pioneering work of Gregory et al. [10], researchers have investigated the coating methods or chemical deposition of aniline on PET fabric or fiber substrate to produce conductive fabric [10,11,12,13,14,15,16,17,18,19,20,21,22,23]. The preparation methods mentioned above are all chemical or electrochemical methods of post-treatment to prepare conductive PANI-coated fibers or fabrics, which will produce toxic wastewater. In recent years, increasing concern about environmental pollution has limited the wide industrial application of chemical surface treatments [24,25,26].

Low-temperature plasma techniques have been increasingly used to induce the polymerization of aniline, owing to a drastic reduction in pollutants [27], free solvents, and the short duration of treatment [12,24,28]. Various plasma deposition methods, including alternating current (AC) plasma [29] and radio frequency (RF) plasma [30,31,32], have been discussed by various authors. However, the majority of plasma polymerizations operate under high vacuum [33], which limits the flexibility of plasma modification [34]. Additionally, some works have been done on the synthesis of PANI films via atmospheric pressure plasma jet, using an inert gas as plasma gas, such as He [35] and Ar [36]. However, few research works have reported using atmospheric pressure dielectric-barrier discharge (DBD) plasma to synthesize PANI coating.

In this paper, we have prepared HCl-doped PANI conductive PET yarns through air DBD plasma treatment under atmospheric pressure, which involves pre-applying aniline and HCl vapors on PET surface and subsequent plasma treatment of the coated yarn. The preparation process is continuous, simple, and clean with easy operation, less consumption and waste of reagents, and no waste water. After the treatment, PANI/PET yarn possessed excellent conductivity. Additionally, HCl-doped PANI coating could improve the wettability of substrate significantly due to the N-containing polar species in PANI [37]. Based on our previous work [38,39], the oxygen- and nitrogen-containing polar groups grafted on the PET yarn surface by plasma mainly caused the rise in air drag through forming H-bonds with the water molecules in the air jet. Taking these into consideration, the PANI coating might also increase the air drag of PET yarn. In this study, we have for the first time researched the effect of HCl-doped PANI coating on air drag in a PET yarn sample. The study revealed that, with the increase of coating amount of PANI, the air drag of PET yarns gradually improved. The results obtained provide a new route for the online preparation of conductive PET fabric via air-jet loom, and the increase of air drag of PET weft yarn might improve the efficiency of air-jet loom.

## 2. Materials and Methods 

### 2.1. Experimental Setup and DBD Plasma Treatment Details

**Figure 1:**
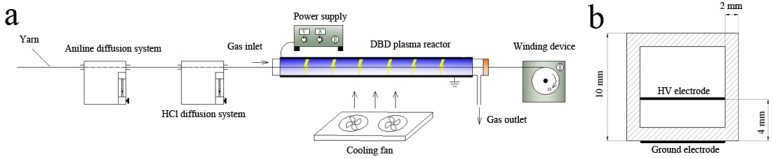
(**a**) Experimental device of vapor phase in situ polymerization of aniline; (**b**) cross section view of electrode configuration.

Figure 1a shows the schematic diagram of DBD plasma treatment platform, consisting of a DBD plasma reactor, a high-frequency AC power supply (CTP-2000K, Coronalab, Nanjing Suman Electronic Co., Ltd., Nanjing, China), aniline vapor diffusion system, an HCl vapor diffusion system, and a winding device (speed adjustable). The DBD plasma reactor was a quartz square tube 300 mm long, 10 mm wide, and 2 mm thick, which can be used to generate DBD in open air under ambient conditions. The discharge gap of the reactor was 4 mm. Stainless fine wire meshes (300 mm in length) were used as high-voltage (HV) and ground electrodes, placed as shown in Figure 1b. An induced draft fan was connected to the gas outlet and a sealed cap with a small circular opening in the center was fixed at the right side of the quartz tube, ensuring the fresh air outside could get into the tube from the gas inlet.

The PET yarn was first pulled by the winding device through the aniline and HCl vapor diffusion systems successively, and afterwards through the DBD plasma reactor to complete the polymerization. The aniline and HCl vapor diffusion rates were controlled by calibrated gas flow meters to adjust the coating amount. The discharge power (*W*) can be calculated by the following equation:

(1) $$W_{p}=UI\cos\theta ,$$

where *U* (V) is the control voltage and *I* (A) is the control current, which can be read directly from the voltage and current meter of AC power supply; θ represents the phase difference between *U* and *I*, which is 45° in the present experiment. The treatment power ranged from 20 W (the lowest power for stable discharge) to 25 W with an interval of 1 W. When the power exceeded 25 W, the PET yarn was seriously ablated. The treatment time, whether 1.5, 2, 3 s, 4.5, or 6 s, was determined by the moving speed of PET yarn and adjusted by the winding device. Liquid aniline monomer was vaporized by means of a bubbler [36], which was supplied by the nitrogen gas (99.999%) with a flow rate ranging from 15 to 75 L/h with an interval of 15 L/h. The diffusion rate of HCl vapor was fixed at a certain value to ensure that the pH value of the PET yarn surface, indicated by moist pH test strips, could be below 3. Under each treatment condition, three copies were prepared. Before various characterizations, the samples were rinsed in an ethanol-water solution (1:1 volume ratio) for 30 min and dried at room temperature.

### 2.2. Materials and Reagents

PET sample yarn was purchased from Ruisheng Fiber Company, Wuxi, China, of which the characteristics was 166dtex/288f. The aniline (C_6_H_5_NH_2_) and hydrochloric acid (HCl) used in this study, of analytical grade, were purchased from Sinopharm Chemical Reagent Co., Ltd., Shanghai, China.

### 2.3. Yarn Conductivity Test

The electrical resistance *R* (MΩ) of yarn samples was measured with a two-electrode system at 20 ± 2 °C and 62~68% relative humidity, according to the testing method for specific resistance of synthetic staple fibers (GB/T 14342-93). Each sample yarn, 2 m in length, was compressed into a tablet under the same pressure in a glass sample tube with an inner diameter of 6 mm. The volume of each tablet was constant. Each sample was measured five times. The resistance was gauged by a digital insulation megger (UT513-2671, Wuhan Guoyi Technology Co. Ltd., Wuhan, China) when the value was above 1 MΩ, and by a multimeter (15B, Fluke, Everett, WA, USA) when the value was below 1 MΩ. Volume resistivity, *ρ* (MΩ·cm), was calculated by the following equation:

(2) $$\rho =\frac{RS}{L}$$

where *R* is the resistance value, *S* is the base area of the tablet, and *L* is the length of the tablet.

### 2.4. Air Drag Measurement

The air drag of yarn was characterized by the self-designed testing platform, which was composed of an air-supply system and a yarn tension tester. The detailed measurement procedures can be found in our previous work [38]. The increased percentage of air drag (*P*_d_%) can be expressed by Equation (3):

(3) $$P_{d}\%=\frac{\left(F_{\mathrm{di}}-F_{d0}\right)}{F_{d0}}\times 100\%$$

where *F*_di_ is the air drag of the treated sample and *F*_d0_ is that of the untreated sample.

### 2.5. Yarn Diameter Measurement

**Figure 2:**
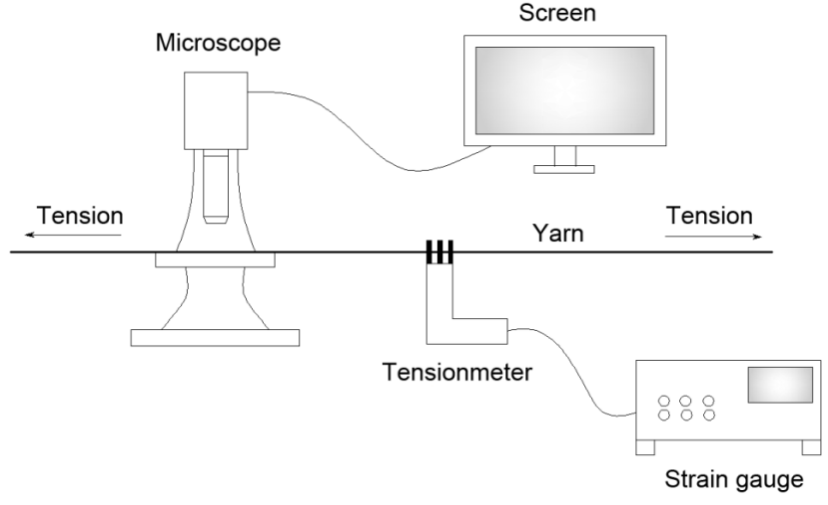
Schematic diagram of yarn diameter measurement setup.

The yarn diameter was measured with a wide-field fluorescence microscope (MacroZoom Z16, Leica, Bannockburn, IL, USA). Owing to the elasticity of the fiber bundle, the yarn diameter varied with the change of tension. Therefore, each yarn sample was applied with tension equal to its air drag, during the diameter measurement (see the schematic diagram in Figure 2). Each sample was prepared as three copies and the measurements were performed at 30 different locations on each copy randomly, of which the average was taken.

### 2.6. Scanning Electron Microscopy (SEM) and Atomic Force Microscopy (AFM)

A field emission scanning electron microscope (S-4800, HITACHI, Tokyo, Japan) was employed to observe the morphology of the samples. Scanning electron microscopy (SEM) analyses were performed using an acceleration voltage of 3 kV. Samples for SEM measurements were coated with Au film using a Sputter Coater (E-1045, HITACHI). The morphology and roughness of the PANI-coated PET fiber surfaces were characterized by an atomic force microscope (Dimension Icon, Bruker Corporation, Billerica, MA, USA).

### 2.7. Fourier Transform-Infrared (FT-IR) Spectroscopy Analyses

Fourier transform-infrared (FT-IR) spectra were collected on a Nicolet 5700 spectrometer (Thermo Electron Corporation, Waltham, MA, USA) using a transmission mode between 4000 and 400 cm^−1^.

### 2.8. X-Ray Photoelectron Spectroscopy (XPS) Analyses

XPS analyses were carried out at a base pressure of 5 × 10^−10^ mbar and a temperature around −100 °C. The XPS spectra were recorded using a Kratos Axis Ultra DLD spectrometer (Manchester, UK) employing a monochromated Al-Ka X-ray source (1486.6 eV). The binding energy (BE) scale was calibrated with reference to the C1s line at 284.5 eV. N1s and C1s high-resolution core level spectra were obtained for the samples of PANI-coated PET yarn.

## 3. Results and Discussion

### 3.1. The Polymerization of PANI via DBD Plasma and the Effect of the Coating Amount of PANI on Volume Resistivity

**Figure 3:**
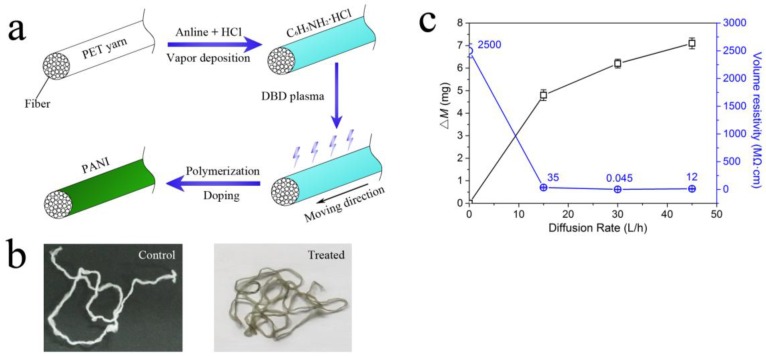
(**a**) Illustration of the treatment procedure; (**b**) chemical structures of idealized oxidation states of PANI; (**c**) the effect of coating amount (Δ*M*) of PANI on volume resistivity of PET yarns.

Figure 3a schematically illustrates the treatment procedure. Firstly, aniline and HCl were successively absorbed by the PET surface through a vapor deposition method, on which an aniline hydrochloride coating was formed. The coated yarn was subsequently subjected to an atmospheric air DBD plasma treatment, inducing the polymerization of aniline and HCl doping of PANI. After the plasma treatment, the online-prepared PET yarn turned conductive. As shown in Figure 3b, the control PET yarn was white, while the sample turned dark green after plasma treatment, indicating the presence of PANI-EB [40]. The volume resistivity of the PET sample treated with optimal experimental condition (22 W, 3 s, 30 L/h) was 0.045 MΩ·cm, approximately five orders of magnitude lower than that of the control one (2500 MΩ·cm). Generally, PANI coatings were prepared by a chemical or electrochemical method using an oxidant as initiator, such as hydrogen peroxide [41] or ammonium persulfate [42]. In this study, the reactive oxygen species (e.g., free radicals and peroxides) in DBD plasma were utilized as oxidants to induce the in situ polymerization of aniline monomers on the surface of moving PET yarn. Figure 3c shows the effect of coating amount (Δ*M*) of PANI on the volume resistivity of PET yarns. Three levels of aniline diffusion rate, 15, 30, and 45 L/h, were chosen to adjust the coating amount. Other treatment conditions of each PET yarn sample, treatment power, and time were the same as those of the optimal sample (22 W, 3 s). The coating amounts (Δ*M*) for the three treated samples were 4.8 mg (15 L/h), 5.9 mg (30 L/h), and 7.1 mg (45 L/h), while the mass of these samples (8 m in length) before the treatment were 130.4, 128.5, and 129.2 mg, respectively. The volume resistivity for the other two treated sample yarns were 35 MΩ·cm (15 L/h) and 12 MΩ·cm (45 L/h). It is implied that, under the same plasma treatment conditions, there is no linear relationship between coating amount and volume resistivity; with a certain coating amount, the PET yarn could have optimum conductivity.

### 3.2. The Effect of Coating Amount of PANI on Air Drag of PET Yarn

Based on our previous work, the grafted polar groups on PET yarns via DBD plasma is an important factor to increase the air drag by increasing the interaction between polar groups and water molecules in an air jet [38]. Similarly, the N-containing polar groups in PANI-EB (e.g., –N^+^H–) might also improve the air drag. Therefore, the effect of coating amount of PANI-EB on air drag has been studied. The samples used were the same as those in Section 3.1.

Table 1 shows the effect of the PANI coating amount on the characteristics of the treated yarns, such as air drag (*F*_d_), yarn diameter (*d*), and coating amount (Δ*M*). With the increase in the aniline diffusion rate, the weight of samples improved gradually, while the diameter varied subtly. The drag force of coated PET yarns obviously improved; the increased percentages (*P*_d_%) were 4.6% (15 L/h), 7.5% (30 L/h), and 12.3% (45 L/h), respectively.

The air drag (*F*_d_) of yarn, which is used to evaluate the weaving efficiency of an air jet, can be expressed by:

(4) $$F_{d}=\frac{1}{2}C_{d}\rho \pi d{\left(V_{f}-V_{y}\right)}^{2}L$$

where *C*_d_ is the drag coefficient, *ρ* is the flow density, *d* is the yarn diameter, *V*_f_ and *V*_y_ are the velocity of the air jet and yarn, respectively, and *L* is the length of yarn immersed in the jet flow.

Based on Equation (4), the drag coefficient *C*_d_, which can be affected by the surface properties of yarn, is expressed by:

(5) $$C_{d}=\frac{2F_{d}}{\rho \pi d{\left(V_{f}-V_{y}\right)}^{2}L}$$

When the air drag of sample yarns was measured under the same conditions (the values of *ρ*, *L*, *U* and *V* were constant), only *C*_d_ and *d* could affect the air drag. Therefore, the ratio of *F*_d_ to *d* is equivalent to *C*_d_, which can be utilized to study the effect of *C*_d_ on air drag after plasma treatment. As can be seen in Table 1, the values of *F*_d_ to *d* for each sample were 0.094 (0 L/h), 0.099 (15 L/h), 0.102 (30 L/h), and 0.106 (45 L/h) cN/μm, respectively, indicating that *C*_d_ (air drag) improved with the increased PANI coating amount (polar species). The results further verify that the change of air drag was mainly due to the change of *C*_d_ [39].

### 3.3. Chemical Characterization of Treated PET Yarn Surfaces

**Figure 4:**
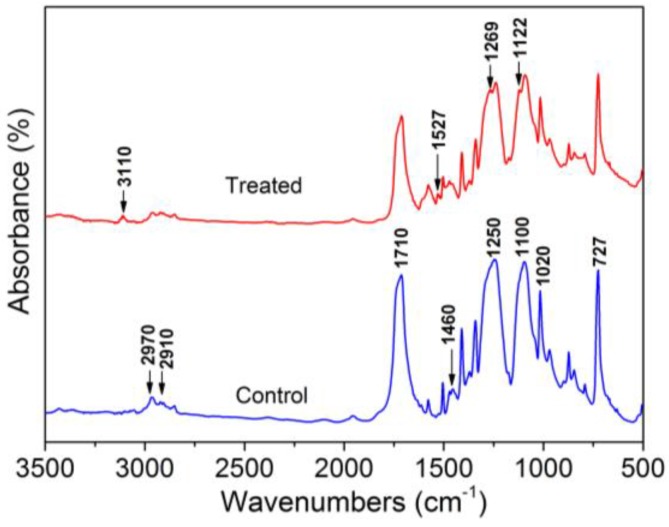
FT-IR spectra of untreated and optimal PANI-coated PET sample yarns.

Figure 4 shows the FT-IR spectra of control and optimal PANI-coated PET sample yarns. As can be seen in the spectrum for control sample, absorption bands at 2970, 2910, and 1710cm^−1^ correspond to the methylene nonsymmetrical stretching vibration, the methylene symmetrical stretching vibration [43], and the C=O symmetric stretching of carbonyl groups [44], respectively. The broad bands at 1250 and 1100 cm^−1^ are mainly attributed to ester C=O stretching [45]. Some bands can be observed in both spectra, attributed to benzene groups from both PANI and the PET substrate, which appear at 727 cm^−1^ (out-of-plane vibration of the benzene group), and 1020 cm^−1^ (in-plane vibration of benzene), and 1460 cm^−1^ (C–C stretching vibration of benzenoid rings) [45]. The characteristic peaks of the emeraldine base form of PANI can be observed in the spectrum for the optimal sample at 3110 cm^−1^ (N–H stretching with hydrogen-bonded 2° amino groups); 1527 cm^−1^ (C–C stretching of quinoid rings); 1269 cm^−1^ (C–N stretching) [46], and 1122 cm^−1^ (N=Q=N bending vibration, with Q denoting a quinoid ring) [47], which further supports the formation of PANI via the plasma polymerization technique.

**Figure 5:**
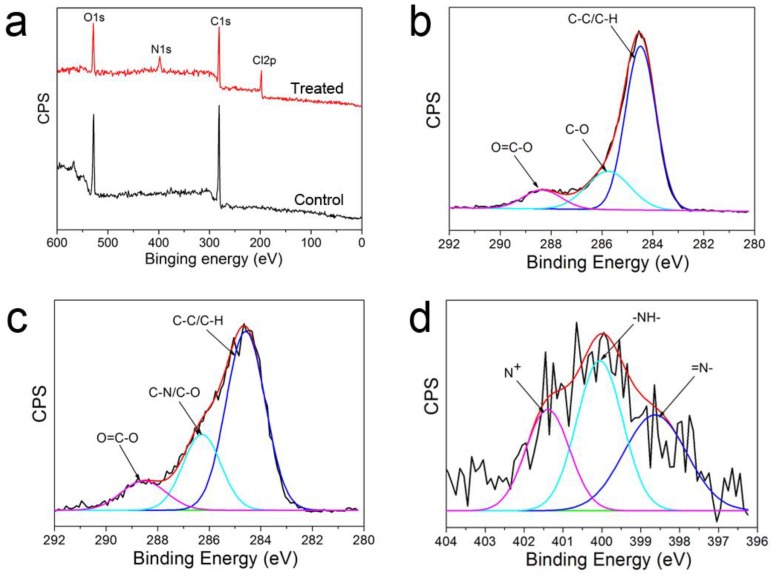
(**a**) XPS wide-scan spectra of PET yarns. High-resolution C1s spectra of (**b**) control and (**c**) optimal PET yarns. (**d**) High-resolution N1s spectra of optimal sample.

XPS analysis of control and treated PET yarn samples has also been performed (Figure 5). As shown in Figure 5a, the elements C1s (at 284.5 eV), N1s (at 398.4 eV), O1s (at 531.8 eV), and Cl2p (at 198.7 eV) were detected on the optimal PANI-coated PET yarn, while on the control PET surface only C and O elements can be detected. The high-resolution XPS spectra of C1s and N1s are used to quantitatively analyze the surface composition (Figure 5b–d). In Figure 5b,c, three peaks at 284.5, 285.8, and 288.4 eV can be observed, corresponding to C–C/C–H, C–O/C–N, and O=C–O, respectively [48,49]. Figure 5d shows the high-resolution N1s spectra for the PANI-EB coating, in which the three peaks centered at 398.6, 399.9, and 401.4 eV are attributed to the quinonoid imine (=N–), bamine-like (–NH–) structure, and positively charged nitrogen atoms (N^+^) [50].

Table 2 shows the quantitative result. As can be seen, the C1s and O1s atomic contents were 75.8% and 24.2%, respectively. After plasma treatment, the C1s and O1s atomic contents decreased slightly to 74.9% and 19.2%. Meanwhile, the new elements, N1s (4.6%) and Cl2p (1.3%), were detected on the treated PET surface; moreover, the percent contribution of the C–O (C–N) group increased slightly, indicating that PANI was grafted on the PET surface via DBD plasma polymerization. XPS characterization was also performed to quantify the amount of counter ion (Cl^−^) and the doping ratio (N^+^/N_Total_) of the optimal PANI-coated yarn. The doping ratio can be calculated by the ratio N^+^/N_Total_, obtaining a value of 0.26, and the atomic concentration of N^+^ was 1.2%. From Table 2, the atomic concentration of Cl2p (1.3%) was close to that of N^+^, which verified the doping efficiency.

### 3.4. Surface Morphology Characterization of Treated PET Yarns

**Figure 6:**
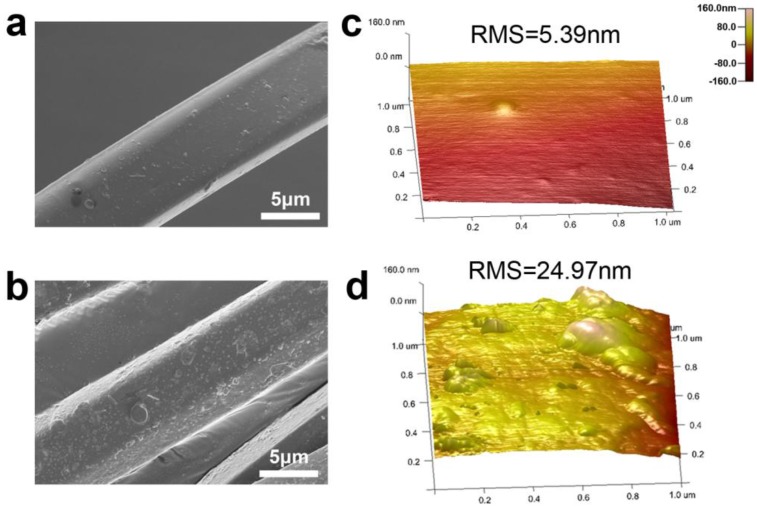
SEM characterization of samples: (**a**) untreated yarn; (**b**) optimal yarn. AFM characterization of samples; (**c**) untreated yarn; (**d**) optimal yarn.

Figure 6 shows scanning electron microscopy (SEM) and atomic force microscopy (AFM) images of PET fiber surfaces before and after plasma treatment. As shown in Figure 6a, the untreated PET has a relatively smooth surface. After plasma treatment, the fiber surface became rougher (Figure 6b), and uniformly distributed protuberances can be observed. The AFM images show the morphology for the untreated and plasma-treated sample surfaces (Figure 6c,d). Based on the AFM images, the root mean square (RMS) roughness was estimated. The RMS roughness of pristine cotton surface is 5.39 nm. After DBD plasma treatment, the roughness increased to 24.97 nm. The formation of a uniform PANI coating on PET fiber surface was mainly attributed to its hydrophobicity, which was explained by the preferential adsorption of the reactive intermediates such as aniline cation radicals and oligomers onto the hydrophobic surface [51].

## 4. Conclusions

In this paper, PANI-coated conductive PET yarn has been successfully prepared through a vapor phase in situ polymerization induced by DBD plasma under atmospheric pressure. The reactive oxygen species in plasma were utilized as oxidants to trigger the polymerization of the aniline monomer. Under optimal plasma treatment conditions (32 V, 3 s, and 30 L/h), the PANI-coated PET yarn possesses excellent conductivity (0.045 MΩ·cm), five orders of magnitude lower than that of the control (2500 MΩ·cm). Moreover, the air drag of coated PET yarns increased with the rise in coating amount of PANI. In summary, this plasma-induced polymerization is continuous, simple, and clean, which could offer a new method to prepare conductive fabrics and is expected to increase the weaving efficiency of an air-jet loom.

## Figures and Tables

**Table 1:** Effect of PANI coating amount on the characteristics of the treated yarns.

Diffusion rate (L/h)	Δ*M* (mg)	*d* (μm)	*F*_d_ (cN)	*F*_d_/*d* (cN/μm)
0	0	251.6	23.87	0.094
15	4.8	252.3	24.88	0.099
30	5.9	251.1	25.57	0.102
45	7.1	252.4	26.69	0.106

**Table 2:** Quantification analysis of elements on control and optimal PET yarns.

Element	Control	Treated
C1s	75.8	74.9
O1s	24.2	19.2
N1s	0	4.6
Cl2p	0	1.3
C1s and N1s groups		
C–H/C–C	67.4	62.6
C–O/C–N	22.9	25.7
O=C–O	9.7	11.7
=N–	0	34.7
–NH–	0	39.7
N^+^	0	25.6
N^+^/N_Total_	-	0.26
